# Light Regulated CoWRKY15 Acts on CoSQS Promoter to Promote Squalene Synthesis in *Camellia oleifera* Seeds

**DOI:** 10.3390/ijms252011134

**Published:** 2024-10-17

**Authors:** Aori Li, Qinhui Du, Yanling Zeng, Rui Yang, Luyao Ge, Ziyan Zhu, Chenyan Li, Xiaofeng Tan

**Affiliations:** 1 Key Laboratory of Cultivation and Protection for Non-Wood Forest Trees of Ministry of Education, Central South University of Forestry and Technology, Changsha 410004, China; 20221100070@csuft.edu.cn (A.L.); 19573147675@163.com (Q.D.); 15700768591@163.com (R.Y.); 15936384085@163.com (L.G.); zzyzzy200011@163.com (Z.Z.);18160580572@163.com (C.L.); 2 Key Laboratory of Non-Wood Forest Products of State Forestry Administration, Central South University of Forestry and Technology, Changsha 410004, China

**Keywords:** *Camellia oleifera*, squalene, squalene synthase (SQS), Yeast one-hybrid screening, CoWRKY transcription factor, expression

## Abstract

Squalene synthase (SQS) is the most direct key enzyme regulating squalene synthesis. To better understand the regulatory mechanisms of squalene biosynthesis, a 1423-bp long promoter region of the *CoSQS* gene was isolated from *Camellia oleifera*. Plant CARE and PLACE analysis affirmed the existence of the core promoter elements such as TATA and CAAT boxes and transcription factor binding sites like W-box and MYB in the isolated sequence. Exogenous factors regulating the *CoSQS* promoter were obtained by using Yeast one-hybrid screening, and the key transcription factor CoWRKY15 was found. AOS (Antibody Optimization System) analysis showed that CoWRKY15 had the highest interactions with a confidence level of 0.9026. Bioinformatics analysis showed that CoWRKY15 belonged to class 2 of the *WRKY* gene family. The results of subcellular localization showed that CoWRKY15 functioned in the nucleus. The results of CoWRKY15 promoter analysis showed that 8 out of 14 cis-elements with annotatable functions were related to the light response. The region of the *CoSQS* promoter that interacts with CoWRKY15 is −186 bp~−536 bp. The histochemical assay and squalene content suggested that the *CoSQS* promoter could drive the expression of GUS gene and specific promotion of *CoSQS* expression. It was found that CoWRKY15 could act on the −186 bp~−536 bp *CoSQS* promoter to regulate the expression of *CoSQS* and the content of squalene in *C. oleifera* seed kernels.

## 1. Introduction

*Camellia oleifera* (Oil Tea), in a broad sense, refers to more than 60 shrubs of the genus Camellia (Theaceae) whose seed kernels produce high-quality edible oils [1]. *Camellia* oil is not only rich in unsaturated fatty acids, but also rich in squalene and other bioactive substances [2]. Squalene is an open-chain triterpene organic substance. Squalene is involved in tumor suppression and immune enhancement, has antibacterial and antiviral activity, and can be used as a drug carrier and adjuvant for vaccines [3]. Although some studies have shown that *C. oleifera* seed kernels are rich in squalene, the mechanism involved is not clear. At present, there are two main synthetic pathways for the biosynthesis of plant triterpene saponins, methyl-d-erythritol-4-phosphate (MEP) and mevalonate (MVA), of which the MVA pathway is the main pathway for the synthesis of plant triterpenoids [4]. Researchers have speculated that the MVA pathway is the main pathway for triterpenoid backbone biosynthesis in *C. oleifera* based on the expression patterns of triterpenoid-related oxidosqualene cyclases (OSCs) [5]. In the MVA pathway, squalene synthase (SQS) is located at the branch point of the plant triterpene synthesis pathway, which is a key rate-limiting enzyme in the upstream pathway of triterpenoid metabolism synthesis. First, farnesyl pyrophosphate (FPP) produces prosqualene pyrophosphate (PSPP) under the action of *SQS*; a second step is followed by a chain reaction: in the presence of nicotinamide adenine dinucleotide phosphate (NADPH), PSPP can be further affected by *SQS* to produce squalene. Comprehensive research progress shows that *SQS* activity and content play a decisive role in the yield of plant triterpene saponins [6,7].

Kim et al. [8] proved that squalene synthase (SQS) regulation is the key factor affecting squalene accumulation as assessed through the *SQS* deletion yeast erg9 mutant functional complementarity test. However, under the same promoter, the ability of SQS mutants to resume squalene synthesis was significantly different after the complementary expression, which indicated the importance of transcription factors. Pirronen V et al. [9] also demonstrated that the most critical influence on squalene accumulation is the transcriptional regulation of the squalene synthase (SQS) gene. Promoters are sequences that initiate and regulate gene transcription. At present, there are more and more reports on the promoters of squalene synthetase genes at home and abroad, such as *Eleutherococcus senticosus*, *Betula platyphylla Sukaczev*, *Ganoderma lucidum*, etc. [10,11,12].

WRKY belongs to the WRKY-GCM1 zinc finger transcription factor superfamily, which is involved in plant growth, development, and physiological responses [13,14]. WRKY proteins in the plant kingdom form a large family of transcription factors that can be divided into at least three groups (Group Ⅰ, Group Ⅱ, and Group Ⅲ) according to the characteristics of the structural domain [15]. The members of the WRKY family Group Ⅰ contain two WRKYGQK domains, and the zinc fingerprint pattern is C-X4-5-C-X22-23H-X-H, such as AtWRKY33 [16] in *Arabidopsis thaliana*. The members of the WRKY family Group Ⅱ contain only one WRKYGQK domain, and the zinc fingerprint pattern is the same as that of the first category, such as GmWRYK13 [17] in soybean. The members of the WRKY family Group Ⅲ contain only one WRKYGQK domain, but the zinc fingerprint is the same. The structural amino acids are changed to C-X7-C-X22-23-H-X-C, such as JrWRKY4 [18] of walnuts.

Yongzhen Sun et al. [15] showed that the expression levels of *HMGR*, *FPS2*, *SQS1*, and *SQE2* were higher than those of the control group after transformation of *PqWRKY1* gene from *P. quinquefolius* into *Arabidopsis thaliana*, and W-box combined with WRKY existed in the promoter regions of the key genes related to triterpenoid synthesis in *A. thaliana* and PqWRKY1 was characterized by structural domains typical of WRKY family class 2 members. Our group compared the changes of squalene content in *C. oleifera* seed kernels during different developmental periods, constructed the transcriptome database, and, based on the analysis that the *CoSQS* promoter contains a W-box cis-element, they retrieved the *CoWRKY1* gene in the transcriptome database, which may interoperate with *CoSQS*. For yeast one-hybrid validation, we preliminarily demonstrated that squalene synthesis in *C. oleifera* seed kernels is affected by the CoWRKY transcription factor. However, the CoWRKY transcription factor is a large gene family [19], and it is not clear whether there are other members that also combine with *CoSQS* to affect squalene synthesis in *C. oleifera* seed kernels.

In this study, the sequence of the promoter of *C. oleifera* squalene synthetase was analyzed by homeopathic element analysis; the foreign factors and transcription factors that may regulate the expression of this gene are preliminarily understood. We combined *C. oleifera* genomic data, constructed *C. oleifera* seed kernel yeast libraries, used the *CoSQS* promoter as bait to regulate transcription factors that may interact with each other, and identified the CoWRKY transcriptional genes with the highest confidence of interaction through AOS (Antibody Optimization System) analysis, based on which we carried out bioinformatics analyses and subcellular localization.

Exogenous factors affecting *CoWRKY* gene expression were predicted through the analysis of its promoter sequence. By truncating pCoSQS and CoWRKY15, Yeast one-hybrid screening was performed to identify the action site of this transcription factor on the *CoSQS* promoter. The biological function of *CoSQS* promoter (pCoSQS) was studied by using tobacco as receptor material. It provides the basis for the further study on the anabolism of aleukiene. The effect of CoWRKY transcription factor on squalene content in oil seeds was further proved, which provided a scientific basis for revealing the function of WRKY transcription factor in oil seeds.

## 2. Results

### 2.1. Bioinformatic Analysis of CoSQS Promoter

Cis element analysis of the 1423 bp sequence in the upstream noncoding region of the *CoSQS* gene using Plant Care, a promoter online analysis database (Table 1). In addition to the conserved elements of the promoter, the promoter shown in the table contains 37 types of cis-acting elements. This gene promoter contains 8 elements associated with light response (GT1-motif, INRNTPSADB, CCA1ATLHCB1, Box 4, ATCT-motif, TBOXATGAPB, SORLREP3AT, ACE), 11 cis-elements involved in hormone response regulation (SEBFCONSSTPR10A, WRKY71OS, ERE, WBOXNTERF3, WBOXATNPR1, NTBBF1ARROLB, GARE1OSREP1, TGACG-motif, ASF1MOTIFCAMV, AR), and FAT related hormones including auxin, ethylene, salicylic acid, and methyl jasmonate. There were 7 WRKY transcription factor binding sites for WRKY71OS, and 5 WRKY DNA-binding proteins induced by salicylic acid (SA) specifically recognized WBOXATNPR1. There were 10 MYB transcription factor binding sites. These elements on the promoter of the *CoSQS* gene in *C. oleifera* indicate that the gene expression may be regulated by photosystem and hormone-like signaling substances.

Two anaerobic induction elements (ANAERO1CONSENSUS, ARE) and seven defense and stress response related elements (myc, GT1MOTIFPSRBCS, EECCRCAH1, TC-rich repeats, STRE, MYB, WUN-motif) were also found. These analysis results indicated that under abiotic stress, the corresponding transcription factors would bind to the corresponding binding sites of the *CoSQS* promoter and affect the expression of *CoSQS*.

### 2.2. Yeast One-Hybrid Screening Analyse of CoSQS Promoter as Bait

We performed colony PCR testing on the positive clones obtained from the yeast screening library and performed next-generation sequencing (NGS) sequencing on the PCR products. The sequencing results obtained a total of 478 transcription factor genes. A total of 63 transcription factors were selected for further study, obtaining transcription factors related to ethylene response, MYB family, phosphorus signal response transcription factors, heat response, WRKY family, auxin response factors (ARFs), Squamosa promoter-binding-like protein (SPL7), common plant regulatory factors (GBF3), and GATA transcription factors (Table 2).

**Figure 1:**
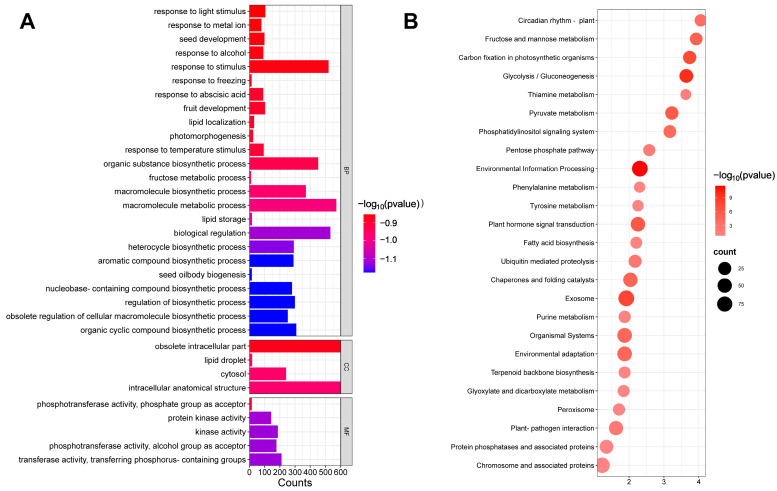
Gene enrichment analysis of *pCoSQS* yeast single hybrid interaction. (**A**) Functional enrichment analysis of GO genes. The longer the horizontal axis represents the number, the greater the number of genes enriched to this function, and the redder the column color, the more significant it is. (**B**) KEGG enrichment pathway analysis. The dot represents the larger the number of origins, the greater the number of genes from the negative electrode to this pathway, and the redder the origin, the more significant it is.

Using CoSQS-QDZ motifs constructed on the pHIS2 vector as bait, a yeast single hybrid *C. oleifera* library was screened. After multiple reporter gene detection, DNA sequencing, and BLAST alignment analysis of positive clones, the proteins interacting with pHIS2 CoSQS-QDZ were identified. GO gene functional enrichment analysis was performed on the sequenced genes (Figure 1A), and the genes that interacted with *CoSQS* promoter were mainly enriched in biological processes (BP), including seed oil generation, lipid storage, as well as genes related to light stimulation and abscisic acid response (seed oilbody biogenesis, lipid storage, response to abscisic acid, response to light stimulus). The genes that interact with the *CoSQS* promoter were also involved in regulating transcriptional activity and kinase activity in terms of molecular function (MF). The relatively small proportion of cellular components (CC) was mainly due to genes related to cellular basic functions. We then identified the number of genes related to a specific pathway through KEGG enrichment analysis on the sequenced gene set. A total of 28 pathways were screened for KEGG enrichment pathway analysis (Figure 1B). Combining *CoSQS* promoter cis acting elements analysis, the expression of *SQS* gene in *C. oleifera* might be regulated by photosystem and hormone signaling substances, as well as the synthesis of terpenoids related to *SQS* gene expression. The results showed three pathways: carbon fixation in photosynthetic organisms, plant hormone signaling, and terpenoid backbone biosynthesis. The number of genes enriched in the three pathways were 29, 53, and 11, respectively.

### 2.3. Obtaining WRKY Transcription Factors Based on the CoSQS Promoter Yeast One-Hybrid Screen Library

**Figure 2:**
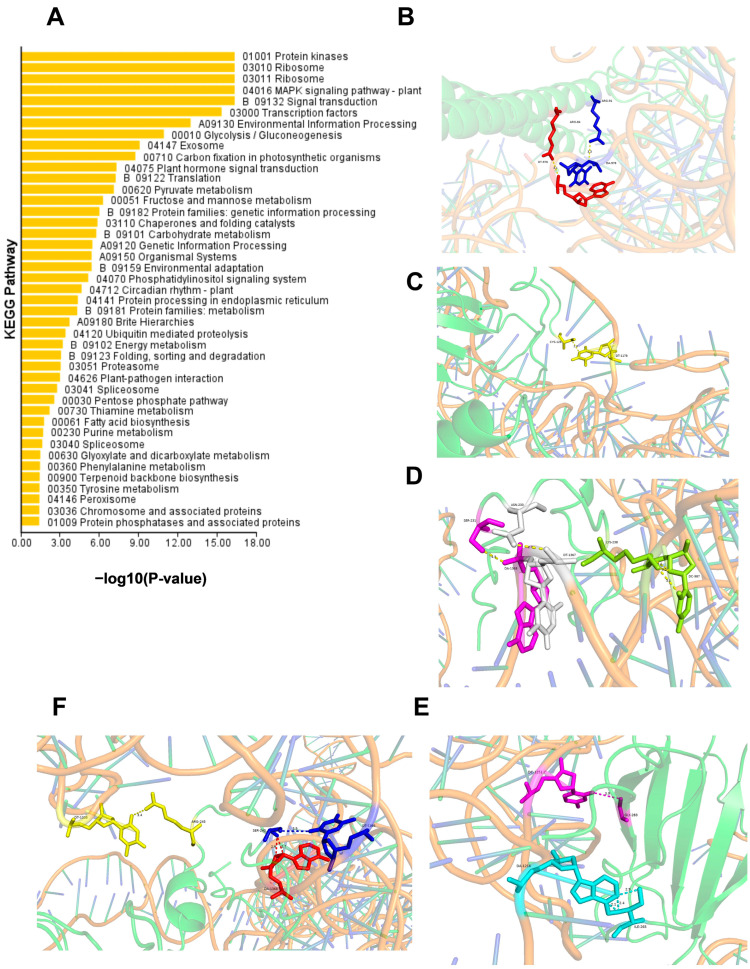
Yeast one-hybrid screen library. (**A**) The metabolic pathways to which NGS belongs; (**B**–**F**) AOS predicted interaction sites between CoWRKY15 and CoSQS promoters. The green color in the background represents the CoWRKY15 protein. The red–blue DNA molecular structure represents the CoSQS promoter. The highlighted part represents different interaction sites between CoWRKY15 and CoSQS promoters.

Based on the *CoSQS* promoter yeast single heterozygous screening library, a total of 1632 NGS sequences were obtained, which were related to 43 metabolic pathways such as signal transduction, environmental adaptation, fatty acid synthesis, and triterpenoid skeleton synthesis, including 379 transcription factors and 394 kinases (Figure 2A). AOS analysis revealed six CoWRKY transcription factors interacting with *CoSQS* promoter and that the CoWRKY transcription factor with the highest similarity to *Camellia sinensis*, WRKY15 [20] (GenBank number: XM_028196157), had the highest confidence in interacting with the *CoSQS* promoter sequence, which was 0.9026. The predicted results of both indicate 11 binding sites: ARG84-DA978, ARG91-DT976, CYS126-DT1178, SER231-DA1368, ASN233-DT1367, LYS238-DC987, SER240-DT1366, ARG245-DT1038, GLY283-DT1251, ILE265-DA1216, SER240-DA1368 (the front represents the CoWRKY protein amino acid position, and the back represents the CoSQS promoter base position) (Figure 2B–F). The WRKY of *C.oleifera* was named *CoWRKY15*.

### 2.4. Bioinformatics Analysis of CoWRKY15

The physical and chemical properties of CoWRKY15 were analyzed using on-line and off-line bioinformatics software. The results showed that the gene encoded 346 amino acids, the theoretical molecular weight (Mw) was 38,297.31 Da, and the isoelectric point (pI) was 9.60, indicating that the protein was alkaline under physiological conditions. The number of negatively charged residues (Asp+Glu) was 33, the number of positively charged residues (Asp+Lys) was 49, and the molecular formula was C1639H2674N508O518S16. The instability index (II) is 52.84 for the typical unstable protein. The total average hydrophobicity (GRAVY) of the protein was −0.616, which was predicted to indicate hydrophilicity.

ProtParam predicted that 9.83% of CoWRKY15 protein was α-helical structure, 11.27% was β-folded, and 78.90% was irregularly coiled with no signal peptide. CoWRKY15 was predicted to function in eukaryotic nuclei with a confidence level of 43. TMHMM predicted the absence of a transmembrane region for the CoWRKY15 protein. The hydrophilic index of CoWRKY15 protein ranged from −3.356 to 1.700, in which hydrophobic residues accounted for 67.34% of the entire amino acid residues, which was typical of hydrophobic proteins. The CoWRKY15 protein existed at one functional site, the WRKY structural domain, located at positions 268–334 Aa, with the amino acid sequence “KMSDIPPDDYSWRKYGQKPIKGSPHPRGYYKCSSVRGCPARKHVERALDDPKMLIVTYEGEHNHSLS”. Judging from the conserved amino acid sequences contained and the structural features of the zinc finger, CoWRKY15 belonged to class 2 members of the WRKY family.

**Figure 3:**
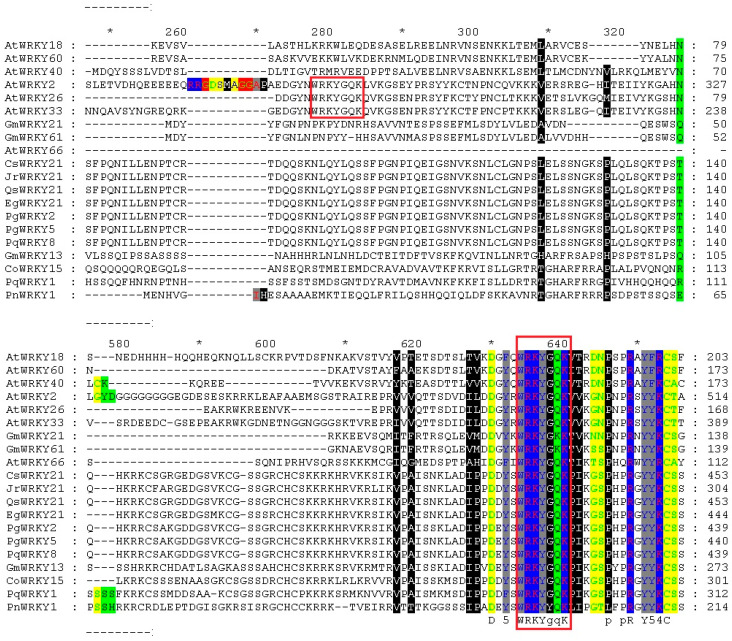
Comparison of amino acid sequences from CoWRKY15 CDS sequence with homologs of other plants WRKY sequences. The red box represents the conservative domain. * indicates consistent sequence.

Based on previous studies [17,21,22] and the results of BLAST on http://www.ncbi.nlm.nih.gov, accessed on 30 May 2023. website, 19 plant WRKY amino acid sequences and CoWRKY15 amino acid sequences were selected for analysis. This phenomenon indicated that the *WRKY* gene family altered greatly during evolution. The conserved domain of the WRKY protein was analyzed and the AtWRKY66 of *A. thaliana* was found to belong to type Ⅲ of the WRKY family. It contains only one WRKYGQK domain and has a typical zinc fingerprint of C-X7-C-X22-23-H-X-C. The AtWRKY26, AtWRKY33, and AtWRKY2 of *A. thaliana* belonged to type Ⅰ of the WRKY family and contain two WRKYGQK domains. The zinc fingerprinting pattern was C-X4-5-C-X22-23H-X-H. The other members belonged to type Ⅱ of the WRKY family with only one WRKYGQK domain (Figure 3). The zinc fingerprinting pattern was C-X4-5-C-X22-23H-X-H. CoWRKY15 of *C. oleifera* belonged to type Ⅱ of the WRKY family ascribed to the typical characteristics of type Ⅱ members.

### 2.5. Analysis of CoWRKY15 Subcellular Localization

**Figure 4:**
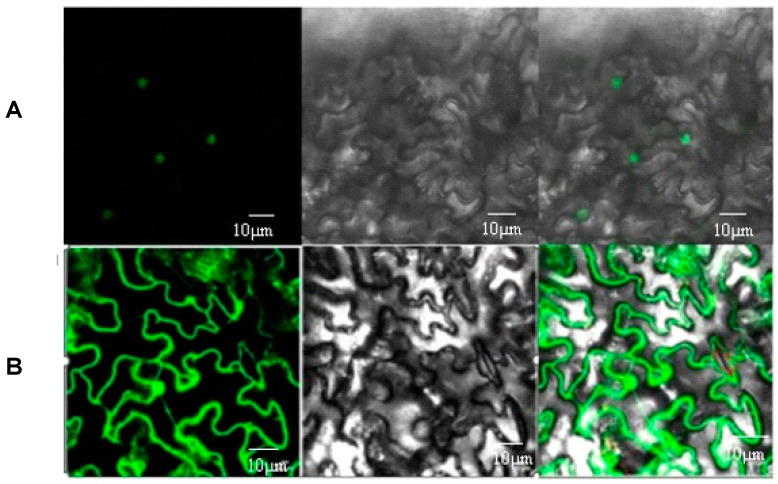
Subcellular localization of CoWRKY15. (**A**) pCAMBIA1300-CoWRKY15-GFP; (**B**) pCAMBIA1300-GFP.

The fusion expression vector was transformed into tobacco leaves by the tobacco injection method, and CoWRKY15 was efficiently transiently expressed in the vicinity of the injected area after 48 h of incubation. eGFP fluorescence signals in tobacco leaves were observed under a confocal laser microscope, and the results showed that eGFP fluorescence signals completely overlapped with autofluorescence signals of the nucleus under the excitation of 543 nm light (Figure 4). This indicated that CoWRKY15 might be located in the nucleus, which is in agreement with the results of the bioinformatics analyses.

### 2.6. CoWRKY15 Expression Related to Squalene Content in C. oleifera Seed Kernels

**Figure 5:**
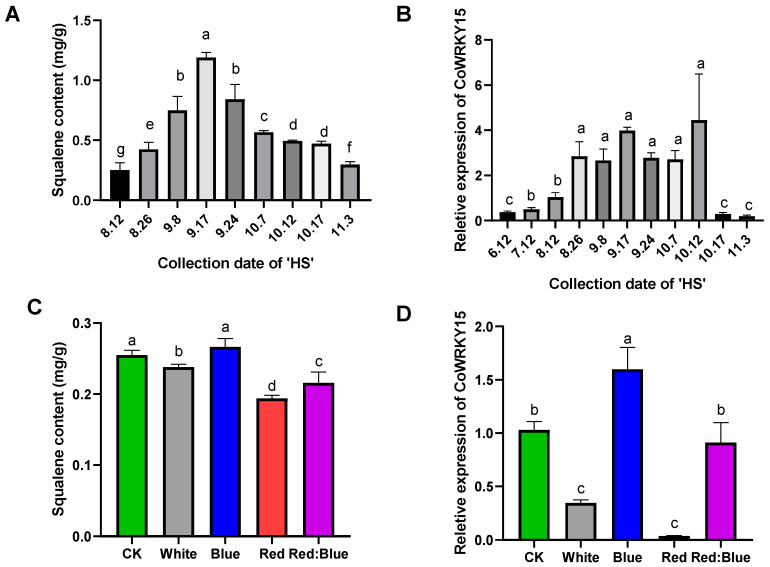
Expression pattern analysis of *CoWRKY15*. (**A**) Squalene content in *C. oleifera* at different developmental stages; (**B**) The relative expression of *CoWRKY15* in different periods of *C. oleifera*; (**C**) Squalene content of *C. oleifera* kernel oil under different light quality conditions; (**D**) *Co WRKY15* relative expression in *C. oleifera* kernels under different light quality conditions. The light quality conditions were as follows: natural light: White, Blue, Red, and Red:Blue = 1:1. The lowercase letters a–g in the histogram represent significant differences; different letters represent significant differences between groups, *p* < 0.05.

Real-Time quantitative PCR results showed that the relative expression of *CoWRKY15* generally showed a trend of first increasing and then decreasing (Figure 5A,B). The expression peak of *CoWRKY15* and squalene content occurred in mid to late September in different developmental stages of *C. oleifera*. This is consistent with the variation trend of squalene content. Correlation analysis was conducted between squalene content and the relative expression level of *CoWRKY15* in *C. oleifera* seeds at different developmental stages. The Pearson coefficient was 0.775 and the significance was 0.014. The results showed that the expression level of *CoWRKY15* was significantly positively correlated with squalene content in *C. oleifera* seeds.

The *CoWRKY15* promoter sequence contained at least eight light-responsive elements (Table S2). Significant differences in the *CoWRKY15* expression and squalene content were observed after different light quality treatments (Figure 5C,D), and bivariate correlation analysis of the two parameters yielded a Pearson correlation of 0.798 and a significance of 0.106.

### 2.7. Interaction Sites and Strength of CoWRKY15 and pCoSQS

**Figure 6:**
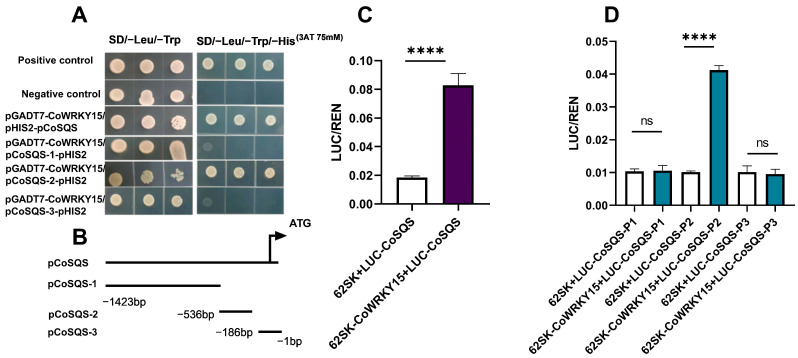
Detection of CoWRKY15 acting at the *CoSQS* promoter. (**A**) Yeast one-hybrid validation; (**B**) Schematic diagram of promoter truncation; (**C**) The effect of CoWRKY15 on the activity of *CoSQS* promoter was determined by dual luciferase assay; (**D**) The effect of CoWRKY15 on the activity of *CoSQS* promoter truncated P1, P2, and P3 was determined by dual luciferase assay. ‘****’: *p* < 0.01.’ns’: *p* > 0.01.

The results of Yeast-one-hybrid showed that all Y187 strains that co-transferred pHIS2 and AD vectors grew well on the SD/-Trp/-Leu medium, which suggested that the co-transformations are successful. The strains of positive control (pGADT7-Rec2 + pHIS2-p53) and the interaction assay were cultured in SD/-Trp/-Leu/-His (3-AT with 75 mM concentration) medium. The positive control and the corresponding experimental groups pHIS2-pCoSQS/pGADT7-CoWRKY15 and PHIS2-PCOSQS-P2/pGADT7-CoWRKY15 grew colonies (Figure 6A), which indicated the interaction between pHIS2-pCoSQS-P2 and pGADT7-CoWRKY15. Experimental group pHIS2-pCoSQS-P1/pGADT7-CoWRKY15 and experimental group pHIS2-pCoSQS-P3/pGADT7-CoWRKY15 did not grow colonies. The results indicated that pHIS2-pCoSQS-P1/pGADT7-CoWRKY15 and pHIS2-pCoSQS-P3/pGADT7-CoWRKY15 had no possibility of interaction. This is consistent with the results predicted by the promoter cis-acting elements, indicating that CoWRKY15 binds to the acting elements at the position of the *CoSQS* promoter −186 bp to −536 bp, where the W-box domain is located.

The results of dual-luciferase showed that the LUC/REN ratios measured after co-transforming 62SK-CoWRKY15 and 0800LUC-pCoSQS expression was about 4 times that of the control, and the difference was extremely significant. Based on the results of *CoSQS* promoter sequence analysis, 1.423 kb *pCoSQS* were cut into three fragments (P1–P3) (Figure 6B) and fused with the pGreen II-0800-LUC report group. The LUC/REN ratios of co-transformed 62SK-CoWRKY15 and 0800LUC-pCoSQS-P2 are close to the LUC/REN ratios of the full length of the *CoSQS* promoter (Figure 6C). However, the LUC/REN ratios of co-transformed 62SK-CoWRKY15 and 0800 LUC-PCOSQS-P1 and 62SK-CoWRKY15 and 0800 LUC-PCOSQS-P3 showed no significant difference compared with the control group (Figure 6D). This indicates that CoWRKY15 can activate the *CoSQS* gene promoter, and it can be further seen that the CoWRKY15 transcriptome interacts with the promoter truncated segment P2.

### 2.8. CoSQS Promoter Function Indentication

**Figure 7:**
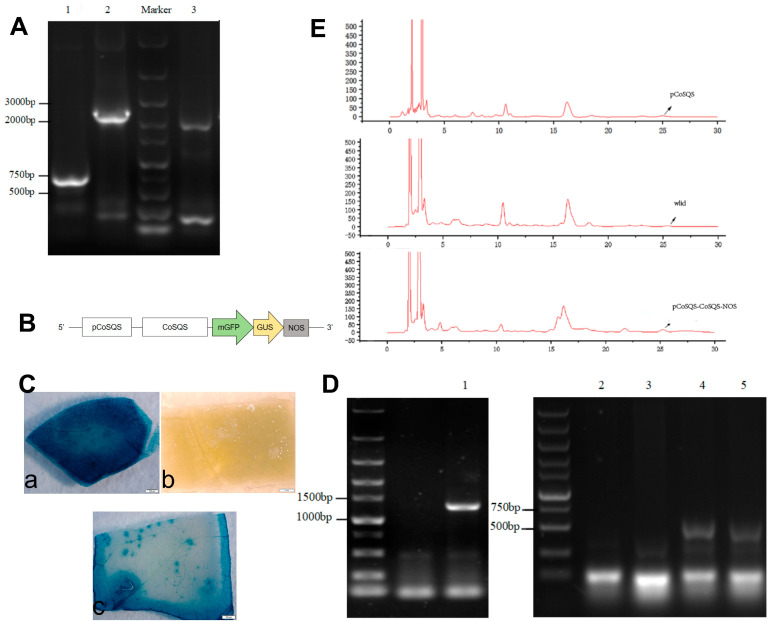
Functional identification of CoSQS promoter transgenic tobacco. (**A**) Universal primer PCR test results (lane 1 is pCAMBIA 1304-*pCoSQS*, lane 2 is pCAMBIA 1304-*pCoSQS-CoSQS-NOS*, lane 3 is pCAMBIA 1304-*pCoSQS-CoSQS*). (**B**) Schematic diagram of 1304-*pCoSQS-CoSQS-NOS* recombinant expression plasmid. (**C**) GUS staining status of T2 generation tobacco leaves (Figure a is the detection result of pCAMBIA 1304-pCoSQS-CoSQS-NOS transgenic tobacco; Figure b is the detection result of wild-type tobacco; Figure c is the detection result of pCAMBIA 1304-pCoSQS transgenic tobacco). (**D**) Tobacco genomic DNA PCR detection results (1 is the detection result of pCAMBIA 1304-pCoSQS-CoSQS-NOS genetically modified tobacco; 2 and 3 are wild-type tobacco detection results; 4 and 5 are the detection results of pCAMBIA 1304-pCoSQS genetically modified tobacco). (**E**) Comparison of squalene content in wild-type tobacco and genetically modified tobacco.

The −600 bp fragment of *CoSQS* (pCoSQS) was amplified from the plasmid of the pHIS2-CoSQS-promoter. The recombinant vectors obtained through continuous homologous recombination using pCAMBIA1304-35S vector were subjected to PCR detection using 1304 universal primers. The detection results of pCAMBIA 1304-pCoSQS, pCAMBIA 1304-pCoSQS-CoSQS, and pCAMBIA1304-pCoSQS-CoSQS-NOS all met expectations (Figure 7A). Through sequencing verification, the pCAMBIA1304-pCoSQS-CoSQS-NOS recombinant expression plasmid was successfully obtained (Figure 7B).

On the 7th day after immersion in the study, callus tissue began to grow on the leaf discs, and on the 27th day, some of the callus tissues successfully grew adventitious buds. Figure S1 shows the tobacco leaf discs on the 9th (a) and 27th (b) day of screening culture medium. We transferred the adventitious buds to the screening medium to induce rooting. We transferred the rooted positive seedlings to the substrate (peat soil: perlite, about 3:1) in order to obtain regenerated plants and to continue to cultivate them for two generations to obtain T2 generation tobacco plants.

We then used GUS staining and DNA PCR detection to detect T2 generation tobacco plants. The T2 generation tobacco leaves were successfully colored and not decolorized after alcohol rinsing, while the control group of wild-type tobacco did not color (Figure 7C). According to the PCR detection results of T2 generation tobacco genome DNA (Figure 7D), the agarose gel electrophoresis bands are in line with the expected pCAMBIA 1304-pCoSQS-CoSQS-NOS, and this shows that the pCAMBIA 1304-pCoSQS recombinant vectors have been successfully transferred into tobacco plants. We then selected tobacco T2 generation plants that passed the testing and continued to cultivate them to obtain T2 generation seeds for subsequent testing.

The HPLC method was used to measure the peak time and peak area of 5 μg/mL, 50 μg/mL, 100 μg/mL, and 150 μg/mL squalene standard samples. A standard curve was plotted based on the peak area and corresponding concentration, and a regression equation was obtained. The fitted curve was Y = 29.595X + 157.5, R2 = 0.9924 > 0.95, indicating high confidence and reliable results. Through HPLC detection and peak analysis related to squalene (Figure 7E), the results showed that the squalene content in T2 seeds of wild-type tobacco, tobacco with only *pCoSQS* transformation, and tobacco with pCOSQS-CoSQS-NOS transformation were 1.948 mg/kg, 0.858 mg/kg, and 94.423 mg/kg, respectively. The significant difference in the results obtained through one-way ANOVA (Table 3) indicate that the content of squalene in tobacco T2 seeds transformed with pCOSQS-CoSQS-NOS is much higher than the first two, indicating that *pCoSQS* has successfully played a role in regulating squalene synthesis in tobacco.

## 3. Discussion

Promoters regulate gene expression through cis-acting element interactions with 30 transcription factors. This study found that the *CoSQS* promoter contains 25 types of cis-acting elements, and the gene promoter also detected 5 elements involved in the photoreaction (ACE, GT1-motif, Box 4, MRE, ATCT-motif). There are 2 cis-related components involved in the regulation of hormone response, such as 1 ethylene response-related element (ERE) and 2 methyl jasmonate response elements (CGTCA-motif, TGACG-motif). Based on the results of yeast single hybrid screening, it can be concluded that the CoSQS promoter interacts with genes related to light stimulation, hormone stimulation, and terpenoid synthesis pathways. Studies have found that methyl jasmonate (MeJA) can induce efficient expression of SQS genes in Panax ginseng and can increase triterpene saponin content [23]. In addition, some SQS genes respond to hormonal and abiotic stresses, for example, the expression of SQS genes in *Bupleurum falcatum* root is significantly upregulated under MeJA, ABA, and ETH treatment [24]. Treatment of *Withania somnifera*, *Glycyrrhiza glabra*, *Tripterygium wilfordii*, *Gardenia jasminoides*, and *Taraxacum mongolicum* with MeJA also upregulated SQS transcription levels [4,25]. In model plant tobacco, squalene synthase (SQS), SE, and oxidized squalene cyclase (OSC) levels are significantly increased after treatment with methyl jasmonate (MeJA) [26]. Combined with the inclusion of one methyl jasmonate response elements (TGACG-motif) in the analysis of *CoSQS* promoter cis-component analysis, it was indicated that the expression of this gene may be regulated by methyl jasmonate, and that MeJA treatment could increase the expression of squalene synthase in *C. oleifera*, thereby regulating the content of squalene in *C. oleifera*.

Exogenous ethylene can promote the biosynthesis of endogenous ethylene during the post-ripening process of *Torreya grandis* ‘Merrillii’, and also promote the expression of relevant genes in the squalene biosynthesis pathway [27]. The results of this study showed that the promoter of the *CoSQS* gene contained ethylene response related elements (ERE), indicating that ethylene may also regulate the expression of *C. oleifera* SQS gene, thereby regulating the synthesis of squalene in *C. oleifera* species.

In addition, the promoter of *C. oleifera* CoSQS gene contains more photosystem and hormonal signaling material response elements, indicating that the content of squalene in *C. oleifera* is regulated by light and that the synthesis of squalene can also be regulated by exogenous hormones.

In addition to these exogenous factors, the cis-acting elements of *CoSQS* promoters also contain the binding sites of several transcription factors (e.g., WRKY71OS, WBOXNTERF3, WBOXATNPR1, MYB). We obtained seven WRKY genes and six MYB genes from the *CoSQS* promoter yeast single hybridized screening library. *C. oleifera* is rich in squalene, which is a pivotal product of the terpene metabolic pathway. Some studies have shown that the key enzyme gene affecting squalene synthesis is *SQS* [28,29]. A previous study by our group found that the expression of *CoSQS* was regulated by the transcription factor WRKY [28]. WRKY is a transcriptional regulator occurring widely throughout the plant kingdom. It participates in regulating the biological and abiotic stresses and physiological responses of various metabolic pathways and is closely related to plant growth, development, and senescence [30,31]. WRKY TFs can specifically recognize the W-box, with sequence TTGAC/T within the target genes’ promoter regions [32]. Hanting Yang et al. [33] also found that WRKY functions as a transcriptional regulator of plant secondary metabolite synthesis. In this study, we used bioinformatics to analyze six WRKY transcription factors that might interact with *CoSQS*, among which the CoWRKY15 transcription factor, which had the highest confidence of interaction, was not the same as the CoWRKY1 transcription factor that was obtained by our group based on transcriptome analysis in the previous stage [31]. Multiple WRKY transcription factors that might affect terpenoid metabolism had also been found in Panax ginseng [34]. The synthesis of terpenoid metabolites could be induced by herbivory, wounding, light, low temperatures, and other stress conditions [35]. It was possible that the WRKY transcription factors that bind to the *CoSQS* promoter were not the same under different conditions, leading to differences in the outcome of transcriptional regulation, which ultimately exhibited differences in squalene content.

According to the number of WRKY domains and the characteristics of a zinc finger structure, WRKY could be divided into Groups I, II, and III. Group II can be further divided into five sub-categories: IIa, IIb, IIc, IId, and IIe [21]. The deduced amino acid sequence of CoWRKY15 gene of *C. oleifera* consisted of a typical WRKY domain and C2H2zinc finger structure, categorized into the second group of WRKY members. Diqiu Yu et al. found that WRKY DNA-binding proteins can recognize and specifically bind to W-box sequences in the promoter region of AtNPR1, which then activates defense gene expression to induce disease resistance. The results of this study also confirmed that CoWRKY15 has a strong transcriptional regulatory function and can bind to the W-box structural domain (WBOXATNPR1:TTGAC) of the *CoSQS* promoter located in the -186 bp~-536 bp region. By analyzing the expression level of CoWRKY15 in the seed kernels of *C. oleifera* at different developmental periods, it was found that CoWRKY15 expression showed a dynamic pattern of up-regulation followed by down-regulation with the maturation of *C. oleifera* kernel, which was consistent with the trend of the changes in the squalene content, and the results of the bivariate correlation analysis showed that the expression of CoWRKY15 was significantly and positively correlated to the squalene content of *C. oleifera* seed kernels. This further illustrated the role of CoWRKY15 in positively regulating squalene synthesis in *C. oleifera*. Notably, the region that interacts with CoWRKY15 contains only one cis-acting element motif (TTGAC) associated with the WRKY transcription factor. This motif is also found in other clips of the *CoSQS* promoter but does not interact with CoWRKY15. It may be that the element at this location is connected to a base sequence (TTGAC-GGGAGCG) that is distinct from the other locations of the element and coincidentally coincides with the methyl jasmonate response element (TGACG-motif) on the promoter cis-element. Cis-element analysis of the CoWRKY15 promoter also revealed a TGACG-motif for methyl jasmonate. This suggests that CoWRKY15 may be influenced by methyl jasmonate and may specifically bind to the *CoSQS* promoter to regulate squalene synthesis.

UV-B treatment of one-year-old potted *C. sinensis* cv. Longjing-43 differentially altered the metabolism of terpenoids with significant effects after 8 h of treatment, demonstrating the strong potential of UV-B application for flavor improvement in tea [36]. This study used different wavelengths of light to treat reproductive period *C. oleifera*, and the results showed that different wavelengths of light also had a significant impact on the content of squalene in *C. oleifera* kernels. The correlation analysis between squalene content in *C. oleifera* seeds and CoWRKY15 expression under different light quality conditions showed a positive correlation between the two, but the correlation did not reach a very significant level. This may be because *CoSQS* directly regulated the synthesis of squalene, while CoWRKY15 affected the squalene content in *C. oleifera* kernels by regulating the expression of *CoSQS*. In addition, bioinformatics software predicts that *CoWRKY15* was a hydrophilic non transmembrane protein located in the nucleus. This study confirmed the predicted results through subcellular localization methods. CoWRKY15 was localized in the nucleus consistent with a location where transcription factors played a role.

**Figure 8:**
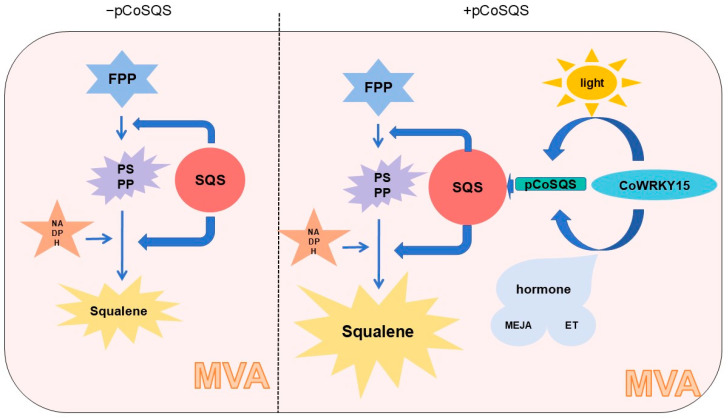
Schematic diagram of MVA synthesis pathway of squalene and pCoSQS expression regulated by transcription factor CoWRKY, light, and hormone.

The promoter is the most important regulatory element; it directly determines the spatiotemporal characteristics and expression intensity of gene expression. In this study, we performed promoter cis-acting element analysis on the pre-promoter 1423 bp of the *CoSQS* gene of camellia oleifera and used yeast single hybridization to screen the *Camellia oleifera* library to obtain the genes interacting with the *CoSQS* promoter of *C. oleifera*. The functional verification of transgenic tobacco was carried out by truncating the *CoSQS* promoter and taking the regions that had interaction with CoWRKY15. It was proved that the *CoSQS* promoter could regulate the squalene synthetase gene to promote the synthesis of squalene in *C. oleifera*. It also further indicated that CoWRKY15 transcription factor could play a role in co-regulating the expression of squalene synthetase gene by influencing the *CoSQS* promoter. The *CoSQS* promoter and CoWRKY15 transcription factor play a regulatory role and are influenced by light and hormones (Figure 8). What is worth further study is that the truncated CoSQS promoter region of camelia oil also contains the binding site of the MYB transcription factor, which may also help pCoSQS regulate the expression of squalene synthetase gene. At the same time, further overexpression of CoWRKY15 transcription factor is needed to verify its function.

## 4. Materials and Methods

### 4.1. Plant Materials

In the gene cloning experiment, the seed kernels of national certified cultivar ‘HS’ were collected from the base of *C. oleifera* in Dongcheng, Hunan, Wangcheng County, Changsha Province. One round of fluorescence real-time PCR detection was carried out on *C. oleifera* ‘Huashuo’ fruits of different developmental stages sampled on 12 June, 12 July, 12 August, 26 August, 8 September, 17 September, 24 September, 7 October, 12 October, 17 October, and 3 November. The squalene content was evaluated in fruits picked on 12 August, 26 August, 8 September, 17 September, 24 September, 7 October, 12 October, 17 October, and 3 November.

Light not only affected the growth of *C. oleifera*, but also had an impact on the metabolism of squalene [18]. The other fluorescent quantitative test materials were *C. oleifera* fruit under different light exposure conditions (Figure S1). Light exposure treatments included blue light (B), white light (W), red light (R), a red–blue light combination (RB; red–blue ratio: 1:1), and sunlight (CK). LED lights were used as light sources for treatment except for CK, including blue (450–480 nm), red (610–640 nm), and white (420–720 nm) LED lights. Spectral characteristics of the different light quality treatments were measured with a HopooColor OHSP-350SF Spectral Color Luminance Meter (HopooColor, Hangzhou, China). The light source was placed above the three-year-old *C. oleifera*, and the height of the LED light source was adjusted to ensure that the photon flux density (PPFD) of each treated plant canopy was 200 ± 10 μmol·m^−2^s^−1^.

Safflower tobaccos were preserved by the Key Laboratory of Cultivation and Protection for Non-Wood Forest Trees of the Ministry of Education, Central South University of Forestry and Technology. Wild-type tobacco (*Nicotiana benthamiana*) was cultivated in nutrient soil and used for genetic transformation experiments. The growth condition was as follows: in the greenhouse under 22 ± 2 °C and 18 h/6 h (day/night) with 45% relative humidity.

### 4.2. Yeast One-Hybrid Screening

Total RNA was extracted from *C. oleifera* kernels using the Trizol method. The mRNA was purified using Oligo (dT) magnetic beads and reverse transcribed into cDNA (Invitrogen, Waltham, MA, USA). After purification, it was homologously recombined with linear pGADT7 and transformed into *Escherichia coli* to construct the *C. oleifera* kernels cDNA library. The 1423 bp upstream sequence of the *C. oleifera* squalene synthase gene (*CoSQS*) promoter was introduced into pHIS2 and transformed into the Y187 strain for background screening to determine the subsequent screening concentration using 75 mM 3AT. The primers used for plasmid construction are shown in Table S1. Using Y187 yeast containing the correct pHIS2-CoSQS-promoter bait plasmid as the receptor strain to prepare the competent cell, the cDNA library plasmid was transferred into it and coated with an SD-TLH + 75 mM 3AT plate. The positive transformants grown on the SD-TLH + 75 mM 3AT screening plate were scraped and subjected to NGS screening and sequencing. Based on the screening library results, KEGG pathway enrichment analysis and GO enrichment analysis were performed.

Based on the bioinformatics analysis of the *CoSQS* promoter, specific primers were used to segment the amplified promoter sequences and construct the vectors proCoSQS-1-pHIS2 (−537 bp to −1423 bp), proCoSQS-2-pHIS2 (−186 bp to −536 bp), and proCoSQS-3-pHIS2 (−1 bp to −185 bp). In addition, specific primers were designed to amplify the full-length cDNA of the CoWRKY15 gene and construct the vector CoWRKY15-pGADT7. The primers are listed in Table S1.

### 4.3. Subcellular Localization

Using the *C. oleifera* cDNA of the test material as template, the products obtained from PCR amplification were recovered and used in the ClonExpress II One Step Cloning Kit (Vazyme, Nanjing, China) to create pCAMBIA1300 using homologous recombination. The primers used for plasmid construction are shown in Table S1.

The healthy and suitable *N. benthamiana* leaves were selected. *A. tumefaciens* containing recombinant plasmids were injected into *N. benthamiana* leaves. The leaves were cultivated for 48 h, and we then observed the fluorescence signal of eGFP under a laser confocal microscope excitation wavelength of 488 nm [37].

### 4.4. Real-Time PCR Quantification of Gene Expression

RNA was extracted from different materials of *C. oleifera* and cDNA was obtained by reverse transcription. The cDNA was then used as a template for real-time PCR to determine the relative expression levels of *CoWRKY15*. *CoEF1α* was used as the internal reference gene [38]. The primers are shown in Table S1. The SYBR Green Kit (TaKaRa Corp., San Jose, CA, USA) for quantitative real-time polymerase chain reaction (qPCR) was applied to CFX96TM real-time PCR detection system according to the manufacturer’s instructions.

### 4.5. Dual-Luciferase Assay

The full-length CDS of the *CoWRKY15* gene was cloned into pGreenII 0029 62-SK, while the promoter sequence of the *CoSQS* gene was inserted into pGreenII 0800-LUC. The primers used for plasmid construction are shown in Table S1. Both constructs were individually transformed into *A. tumefaciens* GV3101 using the freeze–thaw method. Agrobacterium infiltration was carried out with a needle-free syringe. Firefly luciferase and Renilla luciferase activities were analyzed 72 h after infiltration using the Dual-Luciferase Reporter Assay System (DL101, Vazyme, Nanjing, China) with Modulus Luminometers (GloMax96, Promega, Madison, WI, USA). Both luciferase activities were analyzed in three independent experiments with at least six biological replications for each assay.

### 4.6. Construction of Promoter-GUS Fusion Plasmid and Yeast One-Hybrid Promoter Vector

The genomic DNA was extracted from *C. oleifera*’s leaves by using the Ezup Spin Column Super Plant Genomic DNA Extraction Kit (Sangon, Shanghai, China). The quantity and quality of the genomic DNA were measured using a Nanodrop One spectrophotometer (Thermo Fisher, Waltham, MA, USA) and DNA agarose gel electrophoresis.

According to the results of Yeast-one-hybrid and dual-luciferase, the proCoSQS-2 fragment of *CoSQS* promoter could bind to CoWRKY15. At the same time, the proCoSQS-3 part was retained to avoid the destruction of the basic functions of the promoter. Therefore, we isolated 600 bp *CoSQS* promoter fragment (−1 bp to −600 bp) to analyze the function. We designed *pCoSQS* homologous primers and homologous recombination primers using PrimerPremier 5.0 based on the plasmid of pHIS2-CoSQS-promoter (Table S1). We used the Primestar GXL Premix (Taraka Bio, Beijing, China) to clone the promoter. Based on this, homologous recombination ligands were added and cloning vectors were constructed and sent to Qingke Biotechnology (Beijing, China) for sequencing. Use NOS terminator as template, amplify with reference to NOS1304-f and NOS1304-r primers in Table S1, and use 1% agarose gel electrophoresis to recover for standby. Based on cDNA obtained by reverse transcription, use CoSQS1304-BamH1 F in Table S1 and CoSQS1304-KpnI R primers were used to amplify the *CoSQS* gene CDS fragment.

Construct the pCAMBIA 1304-pCoSQS-CoSQS-NOS expression vector using homologous recombination method with reference to the homologous recombination primers in Table S1. Replace the 35 s promoter in the pCAMBIA 1304-35s plasmid with *pCoSQS* and bind to the *GUS* gene. Based on the pCAMBIA 1304-pCoSQS plasmid constructed in the previous step, the pCAMBIA 1304-pCoSQS-CoSQS plasmid was constructed using BamHI and KpnI as restriction endonucleases. Construct the pCAMBIA1304-pCoSQS-CoSQS-NOS plasmid using Sac I and EcoR I restriction endonucleases based on the pCAMBIA 1304-pCoSQS-CoSQS plasmid.

### 4.7. Transformation in Tobacco and Transformant Screening

Follow aseptic procedures to prepare GV3101 Agrobacterium competent cells and transform them into Agrobacterium using an electric shock transformation instrument. Agrobacterium tumefaciens with pCAMBIA1304-pCoSQS plasmid and pCAMBIA1304-pCoSQS-CoSQS-NOS plasmid were transformed into sterile wild-type tobacco seedlings using the leaf disc method. The T0 generation seeds harvested from transformed tobacco were screened and cultivated through two generations to obtain T2 generation plants. When the T2 generation plants grew to 3–4 true leaves, GUS staining and PCR identification were performed. GUS staining method was performed as followed: the transformed tobacco leaves were immersed in GUS staining solution in a 2 mL centrifuge tube for incubation at 37 °C without light. After incubation for 12 h, the explants were placed in ethanol solution with a gradient concentration from 70% to 95% to remove chlorophyll. After decolorization, the images of the explants were taken using the stereomicroscope.

### 4.8. Squalene Content Analysis

*Camellia* oil was extracted using Soxhlet extraction method [39]. Liquid chromatography (HPLC) was used to determine the squalene content in Camellia oil. The detection conditions were as follows: Agilent reverse C18 column, mobile phase (methanol:acetonitrile = 35:65), sample injection volume of 20 μL, flow rate of 1 mL/min, column temperature of 40 °C, and detection wavelength of 210 nm.

### 4.9. Data Analysis

Referring to the genome sequence of *C. oleifera* [1,40], the sequence search was performed using TBtools software (v2.095). The 1423 bp noncoding region sequence upstream of *CoSQS* was intercepted using the Plant Care database (http://bioinformatics.psb.ugent.be/webtools/plantcare/html/, accessed on 1 May 2023). and the online website PLACE (https://www.dna.affrc.go.jp/PLACE/?action=newplace, accessed on 1 May 2023) Promoter cis component analysis.

Interaction prediction was performed using AOS (Antibody Optimization System) by Alphafold (V2.3) and HDock (V1.1), a software developed by Pronetbio Co (Nanjing, China) [41]. The physical and chemical properties and structural characteristics of CoWRKY15 were predicted by on-line software ProtParam (https://web.expasy.org/protparam, accessed on 20 May 2023) and TMHMM (https://services.healthtech.dtu.dk/services/TMHMM-2.0, accessed on 23 May 2023). *CoWRKY15* promoter cis-acting elements were predicted by PLACE (https://www.dna.affrc.go.jp/PLACE/?action=newplace, accessed on 1 August 2023).

Fluorescence quantitative PCR (real-time PCR) data were analyzed by Bio-Rad CFX Manager software (3.1.1621.826). The correlation was analyzed by SPSS Statistics22.0 software.

## 5. Conclusions

In this study, the *CoSQS* promoter was cloned and analyzed from *C. oleifera*. The *CoSQS* promoter had various cis-regulation elements, which included WRKY and MYB binding elements. These transcription factors are bound to *CoSQS* promoter with at least three binding sites. *CoSQS* promoter could promote the expression of *CoSQS*, thereby increasing the squalene content in *C. oleifera* seeds.

CoWRKY transcription factors were present in *C. oleifera* seed kernel in the form of a multigene family, among which CoWRKY15 interacts with *CoSQS* with the highest predicted confidence. CoWRKY15 belonged to the second class of members in the WRKY family, encoding 346 aa, which was a hydrophilic protein without transmembrane structure of about 38.2 kD in size, and it functioned in the nucleus. CoWRKY15 regulated the expression of *CoSQS* mainly by binding to the W-box domain in the −186 bp~−536 bp region of *CoSQS* promoter, and then positively affects squalene biosynthesis in the seed kernel of *C. oleifera*. Appropriate light waves can promote the expression of CoWRKY15 and then enhance squalene synthesis. Appropriate light waves have the effect of promoting CoWRKY15 expression and subsequently increasing squalene synthesis.

## Figures and Tables

**Table 1:** *CoSQS* promoter cis-regulatory element analysis.

Cis-Regulatory Element	Core Sequence	SQS Quantity	Position	Function
GT1-motif	GRWAAW	8	46(−), 79(−), 104(−), 318(−), 1053(−), 1277(+), 1364(+), 1419(+)	Consensus GT-1 binding sites in photoregulatory genes can affect the level of SA (salicylic acid)-induced gene expression
INRNTPSADB	YTCANTYY	8	61(+), 554(−), 567(−), 652(−), 1145(+), 1195(+), 1302(+), 1414(−)	Photoresponsive transcription depends on Inr(initiator)
CCA1ATLHCB1	AAMAATCT	2	213(+), 964(+)	CCA1 binding sites are associated with the regulation of photoallergens
Box 4	ATTAAT	2	299(+), 863(−)	Photoresponsive element
ATCT-motif	AATCTAATCC	1	454(+)	Part of a conserved DNA module involved in photoresponsiveness
TBOXATGAPB	ACTTTG	1	1084(−)	Mutations in Tbox light-activated gene transcription; GAPB encodes the chloroplast glyceraldehyde-3-phosphate dehydrogenase of A.T.
SORLREP3AT	TGTATATAT	1	1227(−)	Photosuppression-related elements
ACE	GACACGTATG	1	1257(−)	Participate in light responsiveness
SEBFCONSSTPR10A	YTGTCWC	2	24(+), 1191(+)	Auxin response element
WRKY71OS	TGAC	7	26(−), 441(−), 539(+), 608(+), 645(+), 722(+), 1118(−)	Transcription suppressor of gibberellin signaling pathway; WRKY protein specific binding site
ERE	ATTTTAAA	9	33(+), 141(+), 307(−), 386(−), 392(+), 910(+), 920(+), 1134(+), 11,369(+)	Ethylene response-related elements
WBOXNTERF3	TGACY	3	440(−), 608(+), 722(+)	W-box element binding site
WBOXATNPR1	TTGAC	5	441(−), 607(+), 644(+), 721(+), 1118(−)	Wbox is specifically recognized by the WRKYDNA-binding protein induced by salicylic acid (SA)
NTBBF1ARROLB	ACTTTA	2	904(+), 1001(+)	Necessary for tissue-specific expression and auxin induction
GARE1OSREP1	TAACAGA	1	1075(+)	Gibberellin reaction element (GARE)
TGACG-motif	TGACG	1	1116(−)	Methyl jasmonate response element
ASF1MOTIFCAMV	TGACG	1	1117(−)	Involved in transcriptional activation of multiple genes by auxin and/or salicylic acid; It may have something to do with light regulation
ARFAT	TGTCTC	1	1192(+)	ARF (growth hormone response factor) binding site
ANAERO1CONSENSUS	AAACAAA	4	117(−), 413(+), 827(−), 939(+)	Anaerobic induction element
ARE	AAACCA	1	473(+)	Anaerobic induction element
myc	TCTCTTA; CATGTG	2	28(−), 1269(−)	Binding sites involved in drought induction
GT1MOTIFPSRBCS	KWGTGRWAAWRW	2	316(−), 1415(+)	The GT-1 motif, which plays a role in pathogen and salt induction
EECCRCAH1	GANTTNC	4	515(+), 810(+), 1277(−), 1328(+)	Binding site of enhancer element Myb transcription factor LCR1
TC-rich repeats	GTTTTCTTAC	1	668(−)	Cis-acting elements involved in defense and stress response
STRE	AGGGG	2	748(+), 1151(−)	Stress response element
MYB	WAACCA, CCWACC, AACGG, TAACARA	10	474(+), 818(−), 821(+), 986(+), 1043(+), 1075(−), 1075(+), 1104(+), 1362(−), 1363(−)	Binding sites involved in drought induction
WUN-motif	CCATTTCAA	1	1297(+)	Trauma response element

**Table 2:** Transcription factor of *CoSQS* promoter Yeast one-hybrid screening.

Category	Exegesis	Gene ID	Number
ethylene-responsive	AP2-like ethylene-responsive transcription factor AIL5	A_chr04_00247, A_chr04_00296, A_chr04_00341, B_chr04_00370	4
	AP2-like ethylene-responsive transcription factor	A_chr01_00704	1
	Ethylene insensitive 3-like 3 protein	B_chr13_00583	1
	Ethylene insensitive 3-like (EIL1)	B_chr03_01565	1
	Ethylene-responsive transcription factor	A_chr09_02774, A_chr15_00273, B_chr15_01424	3
	Ethylene-responsive transcription factor 3 (ERF7)	B_chr05_02256, B_chr05_02545	2
MYB	Alcohol dehydrogenase transcription factor Myb/SANT-like	B_chr08_01909, C_chr03_01517	2
	MYB family transcription factor	A_chr01_01969, B_chr01_01234, B_chr07_00095, C_chr01_01179	4
	Myb-like DNA-binding domain	A_chr04_02547, A_chr14_01812	2
	Myb/SANT-like DNA-binding domain	A_chr08_01652, B_chr08_00772, B_chr08_00938, C_chr08_01305, C_chr10_01486	5
Phosphorus signal	PHR1-LIKE 1-like	A_chr10_00546, B_chr10_02111, C_chr10_02191	3
Heat stress	Heat Stress Transcription Factor	A_chr11_00340, A_chr13_00433, B_chr12_00922, B_chr12_01339, C_chr11_02153	5
	Heat shock factor	B_chr02_02269, C_chr02_00309, C_chr02_00518	3
	Heat shock factor protein	A_chr12_01035	1
WRKY	WRKY transcription factor (WRKY40)	A_chr04_01805, C_chr04_00931	2
	WRKY Transcription Factor	A_chr04_03080, C_chr02_00551, C_chr02_00782, C_chr04_00013, C_chr04_00391	5
ARFS	Auxin response factors (ARFs) are transcriptional factors that bind specifically to the DNA sequence 5′-TGTCTC-3′ found in the auxin-responsive promoter elements (AuxREs)	A_chr07_00284, A_chr15_01009, B_chr13_00986, C_chr01_01589, C_chr03_02429, C_chr07_00661, C_chr07_00696, C_chr07_00844	8
SPL7	Squamosa promoter-binding-like protein (SPL7)	A_chr09_02589, B_chr09_00115	2
GBF3	Common plant regulatory factor (GBF3)	A_chr03_00021, A_chr03_02444, B_chr03_01385, B_chr03_01448, C_chr03_01135	5
GATA	Transcriptional activator that specifically binds 5′-GATA-3′ or 5′-GAT-3′ motifs within gene promoters (GATA2)	A_chr03_02831, B_chr03_00952, C_chr03_01402	3

**Table 3:** Results of one-way analysis of variance.

(I) Constituencies	(J) Constituencies	(I–J) Average Deviation	Standard Error	Statistical Significance
3	1	92.475000 *	0.000816	0.0001
	2	93.565000 *	0.000816	0.0001

1, 2, and 3 are the control group, the *pCoSQS* conversion group, and the *pCoSQS-CoSQS-NOS* conversion group, respectively.’ *’: *p* < 0.05.

## Data Availability

Data are contained within the article.

## Supplementary Materials

The following supporting information can be downloaded at: https://www.mdpi.com/article/10.3390/ijms252011134/s1.
