# Supplementary material for: Light Regulated CoWRKY15 Acts on CoSQS Promoter to Promote Squalene Synthesis in Camellia oleifera Seeds
Source: Int J Mol Sci. 2024 Oct 17;25(20):11134. doi: 10.3390/ijms252011134 (PMC11508267; doi:10.3390/ijms252011134)
Supplement: Supplementary file 1 [file ijms-25-11134-s001.zip › ijms-3245047-supplementary.pdf]

Supplementary Material

Tab S1

Primer name	Primer sequence（5'→3')	usage
CoEF1αF	CTTTCGTCGTTGTCGGCGTTTC	the internal reference gene
CoEF1αR	CAAAGAAGGGTGCCAAGTGA	
pHIS2-pCoSQS-1-F	gccacctcgcccaatggtaccGTTATGTTAGATATTCTAATACATTGTCACATG	
pHIS2-pCoSQS-1-R	tgcgtccggcgtagaggatccTTATAATTTAGATATAATATTTTGTCTTACTAATTTATTT	
pHIS2-pCoSQS-2-F	gccacctcgcccaatggtaccTTATTTTACTTTTTATTTTTAAAAAATTTAAAAA	The decoy fragment was amplified by PCR
pHIS2-pCoSQS-2-R	tgcgtccggcgtagaggatccATAGTGATATATGTGTATATATATATTTATGGGACA	
pHIS2-pCoSQS-3-F	gccacctcgcccaatggtaccATCACTATATCTATCTACATTCGTGTCCG	
pHIS2-pCoSQS-3-R	tgcgtccggcgtagaggatccTTTTTCACTCTGTTCTATCAAAAAAAAC	
pGADT7-CoWRKY15-F	cagctcgagctcgatggatccATGGCCGTCGAGCTCGTG	Homologous recombinant primers were amplified by capture fragment PCR
pGADT7-CoWRKY15-R	gccatggaggccagtgaattcTCAAGAAGACTCTAAGATAAGACCACTTG	
LUC-pCoSQS-F	GTACCGGGCCCCCCCTCGAGGTCGACGGTTATGGTCCCTATTCCTTC	
LUC-pCoSQS-R	GGCTGCAGGAATTCGATATCAAGCTTACGCATAGAAAGAATAGATTG	
LUC-pCoSQS-1-F	ctatagggcgaattgggtaccGTTATGTTAGATATTCTAATACATTGTCACATG	
LUC-pCoSQS-1-R	caggaattcgatatcaagcttTTATAATTTAGATATAATATTTTGTCTTACTAATTTATTT	
LUC-pCoSQS-2-F	ctatagggcgaattgggtaccTTATTTTACTTTTTATTTTTAAAAAATTTAAAAA	Homologous recombinant primers of the dual luciferase test
LUC-pCoSQS-2-R	caggaattcgatatcaagcttATAGTGATATATGTGTATATATATATTTATGGGACA	
LUC-pCoSQS-3-F	ctatagggcgaattgggtaccATCACTATATCTATCTACATTCGTGTCCG	
LUC-pCoSQS-3-R	caggaattcgatatcaagcttTTTTTCACTCTGTTCTATCAAAAAAAAC	
SK-CoWRKY15-F	GGCCGCTCTAGAACTAGTGGATCCATGGATTCTTCTCAAAACTGG	
SK-	ATCGATAAGCTTGATATCGAATTCTCATTCCATTCCAA	

CoWRKY15-R	AAATTGGT	
pET32a-CoWRKY15F	GCCATGGCTGATATCGGATCCATGATGGCCGTCGAGCTCGTGATG	Cloning of homologous recombinant primers of CoWRKY
pET32a-CoWRKY15R	CTCGAGTGCGGCCGCAAGCTTTCAAGAAGACTCTAAGATAAG	
pCAMBIA1300-CoWRKY15F	gatcaagagacaggatccgATGATGGCCGTCGAGCTCGTGATG	The homologous recombinant primers of vector pCAMBIA1300-CoWRKY15 were constructed
pCAMBIA1300-CoWRKY15R	tcaccatcggtgcactagtgTCAAGAAGACTCTAAGATAAG	
CoSQS1304-BamH1 F	CACTGGTACGAAGATCTAGAGGATCCATGGGAAGTTTGGGG	Seed kernel cDNA clone CoSQS reverse primer for homologous recombination
CoSQS1304-KpnI R	ATGATTACGAATTCGAGCTCGGTACCTCACATATTACTTGGTC	Speckernel cDNA clone CoSQS reverse primer for homologous recombination
SQSP-Hind III-F2	GTAAAACGACGGCCAGTGCCAAGCTTCTTCGTTTGGTTAGGTTTGTTTTGC	pCoSQS Forward primer for homologous recombination
SQSP-Xbal I-R2	AGCTCGGTACCCGGGGATCCTCTAGATCTTCGTACCAGTGATATAGGGATTG	pCoSQS Reverse primer for homologous recombination
NOS1304-f	CAAGTAATATGTGAGGTACCGAGCTCGATCGTTCAAACATT	Forward primers for NOS homologous recombination
NOS1304-r	ACAGCTATGACCATGATTACGAATTCGATCTAGTAACATAGATGAC	Reverse primers for NOS homologous recombination

Tab S2 Prediction of cis-acting elements in the *CoWRKY15* promoter.

CATEGORY	EXEGESIS	GENE ID	NUMBER	CATEGORY
A-box	CCGTCC	9(-)/679(+)	Cis-acting regulatory element	2
ABRE	ACGTG	235(+)	Cis-acting elements involved in the abscisic acid responsiveness	1
AE-box	AGAAACAA	1146(-)	Part of a module for light respone	1
ARE	AAACCA	1524(-)	Cis-acting regulatory element essential for the anaerobic induction	1
AT-rich element	ATAGAAATCAA	899(-)/1284(+)	Binding site of AT-rich DNA binding protein (ATBP-1)	2
AT1-motif	AATTATTTTTTATT	1796(-)	Part of a light responsive module	1
Box4	ATTAAT	288(+)/1807(-)/1476(-)968(+)/1618(-)	Part of a conserved DNAmodule involved in light responsiveness	5

CGTCA-motif	CGTCA	1308(-)	cis-acting regulatory element involved in the MeJA-responsiveness	1
G-box	TACGTG	234(+)	cis-acting regulatory element involved in light responsiveness	1
L-box	ATCCCACCTAC	707(+)	Part of a light responsive element	1
LAMP-element	CTTTATCA	143(-)	Part of a light responsive element	1
Sp1	GGGCGG	728(-)/1657(-)/774(-)	Light responsive element	3
TGACG-motif	TGACG	1308(+)	cis-acting regulatory element involved in the MeJA-responsiveness	1
Chs-CMA2a	TCACTTGA	1099(-)	Part of a light responsive element	1

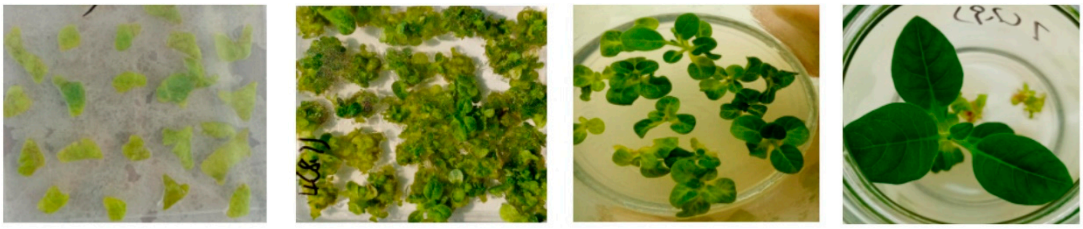

(a) (b) (c) (d)

Figure S1 the tobacco leaf discs on the 9th (a) and 27th (b) day of screening culture medium; Obtained sterile seedlings（c,d）
